# Supplementary material for: XPD stalled on cross-linked DNA provides insight into damage verification
Source: Nat Struct Mol Biol. 2024 May 28;31(10):1580–8. doi: 10.1038/s41594-024-01323-5 (PMC11479942; doi:10.1038/s41594-024-01323-5)
Supplement: Supplementary file 1 — Reporting Summary [file 41594_2024_1323_MOESM1_ESM.pdf]

nature portfolio

Corresponding author(s): Jochen Kuper and Caroline Kisker

Last updated by author(s): Apr 8, 2024

Reporting Summary

Nature Portfolio wishes to improve the reproducibility of the work that we publish. This form provides structure for consistency and transparency in reporting. For further information on Nature Portfolio policies, see our Editorial Policies and the Editorial Policy Checklist.

Statistics

For all statistical analyses, confirm that the following items are present in the figure legend, table legend, main text, or Methods section.

n/a	Confirmed	
☐	☒	The exact sample size (*n*) for each experimental group/condition, given as a discrete number and unit of measurement
☐	☒	A statement on whether measurements were taken from distinct samples or whether the same sample was measured repeatedly
☐	☒	The statistical test(s) used AND whether they are one- or two-sided<br>*Only common tests should be described solely by name; describe more complex techniques in the Methods section.*
☒	☐	A description of all covariates tested
☒	☐	A description of any assumptions or corrections, such as tests of normality and adjustment for multiple comparisons
☐	☒	A full description of the statistical parameters including central tendency (e.g. means) or other basic estimates (e.g. regression coefficient) AND variation (e.g. standard deviation) or associated estimates of uncertainty (e.g. confidence intervals)
☐	☒	For null hypothesis testing, the test statistic (e.g. *F*, *t*, *r*) with confidence intervals, effect sizes, degrees of freedom and *P* value noted<br>*Give P values as exact values whenever suitable.*
☒	☐	For Bayesian analysis, information on the choice of priors and Markov chain Monte Carlo settings
☒	☐	For hierarchical and complex designs, identification of the appropriate level for tests and full reporting of outcomes
☒	☐	Estimates of effect sizes (e.g. Cohen's *d*, Pearson's *r*), indicating how they were calculated

Our web collection on statistics for biologists contains articles on many of the points above.

Software and code

Policy information about availability of computer code

Data collection	EPU software (ThermoFisher) was used for data collection
Data analysis	We have used GraphPadPrism (10.2.1) for data analysis and statistics of biochemical data. For single particle data processing we have used the cryoSPARC suite (4.2.x). For refinement and model building COOT (0.9.8.4), refmac5 (5.8.0352), and the PHENIX suite (1.21rc1-5127) have been used.

For manuscripts utilizing custom algorithms or software that are central to the research but not yet described in published literature, software must be made available to editors and reviewers. We strongly encourage code deposition in a community repository (e.g. GitHub). See the Nature Portfolio guidelines for submitting code & software for further information.

Data

Policy information about availability of data

All manuscripts must include a data availability statement. This statement should provide the following information, where applicable:

- Accession codes, unique identifiers, or web links for publicly available datasets
- A description of any restrictions on data availability
- For clinical datasets or third party data, please ensure that the statement adheres to our policy

Cryo EM data and coordinates have been deposited with EMDB and pdb, respectively. Class 1 and the XPD/DNA complex model are available under the accession

codes EM-19109 and 8rev. Class 2 data are available under EM-19109. Other research data will be made available upon request since the source data for the manuscript have been supplied with the current manuscript. We have used following data from the pdb: 6ro4, 6nmi, and 4q0w.

Research involving human participants, their data, or biological material

Policy information about studies with human participants or human data. See also policy information about sex, gender (identity/presentation), and sexual orientation and race, ethnicity and racism.

Reporting on sex and gender	not applicable
Reporting on race, ethnicity, or other socially relevant groupings	not applicable
Population characteristics	not applicable
Recruitment	not applicable
Ethics oversight	not applicable

Note that full information on the approval of the study protocol must also be provided in the manuscript.

Field-specific reporting

Please select the one below that is the best fit for your research. If you are not sure, read the appropriate sections before making your selection.

☒ Life sciences ☐ Behavioural & social sciences ☐ Ecological, evolutionary & environmental sciences

For a reference copy of the document with all sections, see nature.com/documents/nr-reporting-summary-flat.pdf

Life sciences study design

All studies must disclose on these points even when the disclosure is negative.

Sample size	At least three technical and one biological replicate if not mentioned otherwise. The sample size was determined by intensive pre characterization of the assay systems under various conditions leading to reliable and reproducible results.
Data exclusions	no data was excluded
Replication	all replicates were successful
Randomization	The assay systems were developed by testing a broad range of parameters to ensure reliable results and have been published in many peer reviewed articles
Blinding	Assays were performed independently by different persons yielding comparable results based upon the existing protocols

Reporting for specific materials, systems and methods

We require information from authors about some types of materials, experimental systems and methods used in many studies. Here, indicate whether each material, system or method listed is relevant to your study. If you are not sure if a list item applies to your research, read the appropriate section before selecting a response.

Materials & experimental systems

n/a	Involved in the study	
☒	☐	Antibodies
☐	☒	Eukaryotic cell lines
☒	☐	Palaeontology and archaeology
☒	☐	Animals and other organisms
☒	☐	Clinical data
☒	☐	Dual use research of concern
☒	☐	Plants

Methods

n/a	Involved in the study	
☒	☐	ChIP-seq
☒	☐	Flow cytometry
☒	☐	MRI-based neuroimaging

Eukaryotic cell lines

Policy information about cell lines and Sex and Gender in Research

Cell line source(s)	Insect cell lines were obtained from ThermoFisher
Authentication	Cell lines were authenticated by vendor
Mycoplasma contamination	Cell lines were not tested, since they were only used for protein production. Proteins were then purified to homogeneity and used for assays.
Commonly misidentified lines (See ICLAC register)	not applicable
